# Successful rechallenge with almonertinib and corticosteroids after gefitinib-induced interstitial lung disease: A case report and literature review

**DOI:** 10.1097/MD.0000000000051470

**Published:** 2026-11-06

**Authors:** Jie Liu, Tengchen Wei, Zhenming Bao, Peng Lai, Fangfang Huang, Yunhu Pan

**Affiliations:** a Department of Respiratory and Critical Care Medicine, The 907th Hospital of the PLA Joint Logistics Support Force, Nanping, Fujian, China; b Department of Respiratory and Critical Care Medicine, The 900th Hospital of the PLA Joint Logistics Support Force, Fuzhou, Fujian, China.

**Keywords:** almonertinib, EGFR-TKI, gefitinib, interstitial lung disease, NSCLC

## Abstract

**Rationale:**

First-generation epidermal growth factor receptor (EGFR) tyrosine kinase inhibitors (TKIs) are highly effective in EGFR-mutant non-small cell lung cancer (NSCLC); however, drug-induced interstitial lung disease (ILD) remains a rare but potentially fatal adverse event. The safety and efficacy of EGFR-TKI rechallenge – either with the same agent or an alternative one – following ILD resolution are not established and represent a significant clinical knowledge gap.

**Patient concerns:**

A 53-year-old never-smoking woman with stage IV lung adenocarcinoma – detected to harbor an L858R mutation in exon 21 of the EGFR gene – developed progressive dyspnea and hypoxemia after 2 months of first-line gefitinib therapy, despite confirmed initial radiographic and clinical response.

**Diagnoses:**

The patient was diagnosed with grade 3 gefitinib-associated ILD.

**Interventions:**

With the discontinuation of gefitinib, the patient was given methylprednisolone to resolve ILD. To control advanced lung adenocarcinoma progression, the patient was subjected to 6 cycles of treatment using pemetrexed, carboplatin, and bevacizumab, with measurable tumor shrinkage, followed by maintenance therapy with bevacizumab. Furthermore, given the tumor progression 14 months later, the patient was prescribed docetaxel plus bevacizumab to restore control. Ten months later, the patient experienced disease progression and strongly requested to use EGFR-TKIs again given prior response to EGFR-TKI. The patient was rechallenged with almonertinib, a third-generation EGFR-TKI, and methylprednisolone.

**Outcomes:**

The patient had measurable tumor shrinkage, with sustained tumor control and no recurrence of pulmonary toxicity. As of last follow-up, she maintained progression-free survival for approximately 8 months on almonertinib.

**Lessons:**

This case provides a hypothesis-generating observation, illustrating feasibility only in highly‑selected patients with EGFR‑mutant NSCLC and prior severe TKI‑related ILD. With prophylactic corticosteroids and rigorous surveillance, third‑generation EGFR‑TKI may represent a tentative clinical option for such patients, but general safety cannot be inferred from this single case.

## 1. Introduction

Lung cancer remains the leading cause of cancer-related death worldwide.^[1]^ Non-small cell lung cancer (NSCLC) represents the most common histological type of lung cancer, accounting for up to 85% of all cases.^[2]^ Over the past decades, substantial advances have been achieved in the management of NSCLC, particularly for patients harboring EGFR sensitizing mutations – predominantly exon 19 deletions and exon 21 L858R mutation.^[3]^ Epidermal growth factor receptor (EGFR)-tyrosine kinase inhibitors (TKIs), with well-documented efficacy and favorable safety profiles, are now established as first-line therapy in this population.^[4]^ Nevertheless, EGFR-TKIs may also cause severe adverse events, including interstitial lung disease (ILD), necessitating immediate drug discontinuation.^[5,6]^ Currently, no agreement has yet been reached internationally on the safety or efficacy of TKI rechallenge after ILD. Considering the emergence and increasing application of newer generations of TKIs, rechallenge with an alternative EGFR-TKI after recovery from TKI-related ILD has been accepted as a potential therapeutic strategy.^[7,8]^ This report presents a case of EGFR-mutated lung adenocarcinoma (exon 21 L858R mutation) who successfully transitioned to almonertinib following severe gefitinib-induced ILD, achieving sustained disease control without ILD recurrence.

## 2. Case presentation

A 53-year-old female, with no prior history of hypertension, diabetes, smoking, or alcohol use, and no family history of malignancy, was admitted on August 1, 2022 due to the incidental detection of a pulmonary lesion. Chest computed tomography (CT) on admission revealed a mass in the left upper lobe of approximately 2.6 × 2.2 cm in size (Fig. 1A), accompanied by mediastinal lymphadenopathy and pleural infiltrative nodules, without evidence of ILD (Fig. 2A). With a CT-guided percutaneous lung biopsy, NSCLC was confirmed in this patient following subsequent histopathological examination with hematoxylin and eosin staining. Immunohistochemical analysis showed positive expression of CKpan (+++), TTF-1 (++), NapsinA (++), and Vimentin (+), with negative results for Syn (−), INSM-1 (−), P40 (−); Ki-67 of 3 to 5%, and BRG-1 (+++), supporting a diagnosis of lung adenocarcinoma. Brain magnetic resonance imaging with MR angiography revealed no intracranial abnormalities. Based on the American Joint Committee on Cancer TNM staging system (the 8th edition), the patient was diagnosed with “left lung adenocarcinoma, cT1cN2M1a, stage IVa.” Molecular profiling via next-generation sequencing identified an EGFR exon 21 L858R mutation. On August 10, 2022, the patient initiated oral gefitinib (250 mg daily) as first-line targeted therapy with an ECOG performance status of 1.

**Figure 1.**
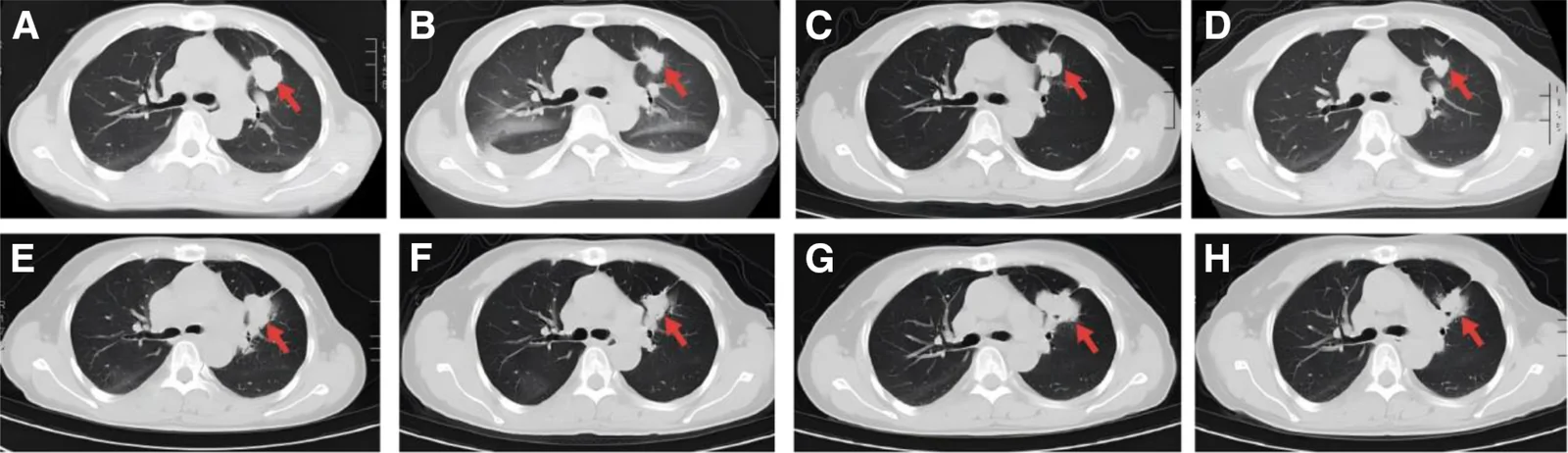
Changes in the lung cancer lesion size on serial chest CT. (A) Baseline (August 1, 2022): 2.6 × 2.2 cm. (B) After 2 months of gefitinib (October 11, 2022): 2.5 × 1.6 cm. (C) After discontinuing gefitinib and approximately 8 weeks of methylprednisolone (December 6, 2022): 2.6 × 1.9 cm. (D) During pemetrexed + carboplatin + bevacizumab treatment (February 28, 2023): 1.9 × 1.5 cm. (E) At progression on bevacizumab maintenance, before the supplementation of docetaxel (June 20, 2024): 3.3 × 2.8 cm. (F) During bevacizumab + docetaxel treatment (January 15, 2025): 2.5 × 2.0 cm. (G) At progression before almonertinib rechallenge (April 28, 2025): 3.7 × 2.9 cm. (H) During almonertinib treatment (December 20, 2025): 2.5 × 2.2 cm.

**Figure 2.**
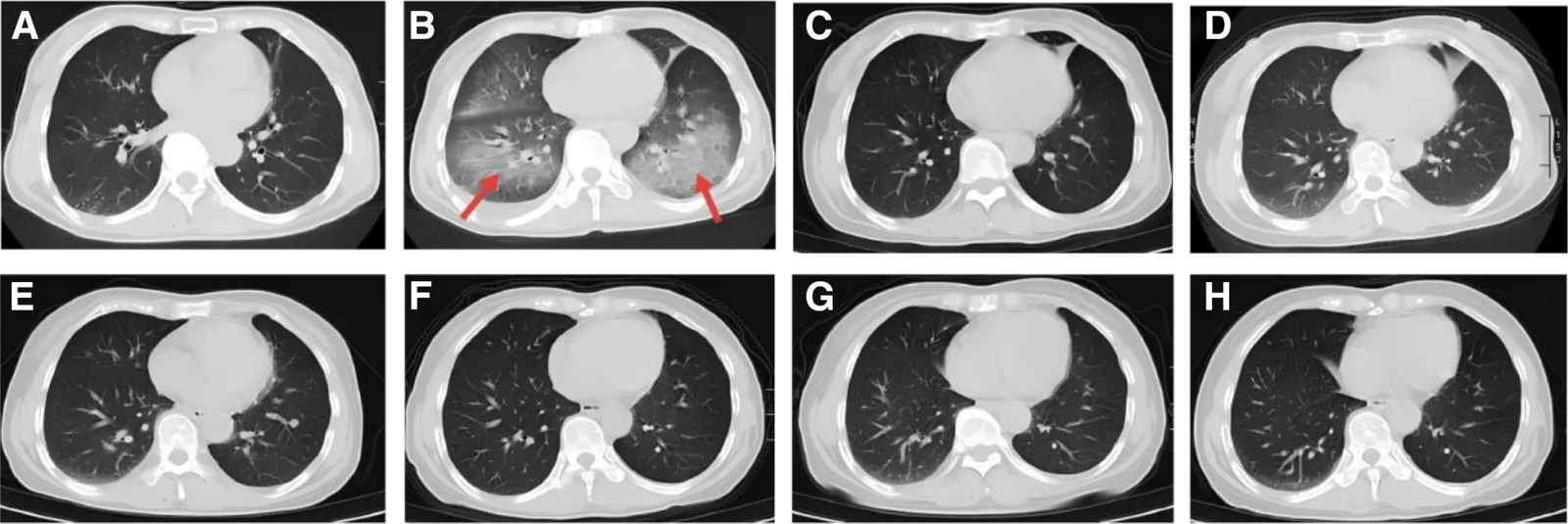
Changes in ILD imaging on serial chest CT. (A) Baseline (August 1, 2022): No definitive imaging evidence of ILD. (B) After 2 months of gefitinib treatment (October 11, 2022): Bilateral pulmonary patchy opacities suggestive of ILD. (C) After discontinuing gefitinib and about 8 weeks of methylprednisolone use (December 6, 2022): Significant ILD improvement. (D) During pemetrexed + carboplatin + bevacizumab treatment (February 28, 2023): No imaging evidence of ILD. (E) At progression on bevacizumab maintenance, before the supplementation of docetaxel (June 20, 2024): No imaging evidence of ILD. (F) During bevacizumab + docetaxel treatment (January 15, 2025): No imaging evidence of ILD. (G) At progression before almonertinib rechallenge (April 28, 2025): No imaging evidence of ILD. (H) During almonertinib treatment (December 20, 2025): No imaging evidence of ILD.

Following 2 months of treatment, the patient was readmitted owing to “cough and shortness of breath.” Arterial blood gas analysis under ambient air conditions revealed a pH of 7.53, a partial pressure of CO_2_ of 33.1 mm Hg, a partial pressure of oxygen of 47 mm Hg, and an oxygen saturation level of 81.3%, suggesting signs of inadequate oxygenation. A chest CT scan on October 11, 2022 indicated a shrinkage of the lung tumor (2.5 × 1.6 cm, Fig. 1B), but with patchy areas of increased attenuation with ill-defined margins in both lungs, particularly in the bilateral lower lobes (Fig. 2B). To establish a diagnosis of exclusion for gefitinib-induced ILD, comprehensive laboratory, microbiological, cardiac, and radiological assessments were performed to rule out alternative etiologies. Blood routine examination indicated the following: leukocyte count of 4.55 × 10^9^/L (reference range: 3.5–9.5 × 10^9^/L), neutrophil count of 3.47 × 10^9^/L (reference range: 1.8–6.3 × 10^9^/L). The level of C-reactive protein (CRP) was 18.0 mg/L (reference range: 0–8.2 mg/L). Procalcitonin (PCT) was within the normal range (reference range: <0.05 ng/mL). Serum 1, 3-β-D-glucan assay (G test) and galactomannan assay (GM test) were both negative. No pathogenic microorganisms were isolated from sputum culture. Echocardiography revealed normal cardiac structure and systolic/diastolic function without signs of heart failure. Serum B-type natriuretic peptide (BNP) was 42 pg/mL (reference range: <100 pg/mL). Plasma D-dimer concentration was 0.23 mg/L (reference range: <0.5 mg/L). At symptom onset, the primary tumor was reduced in size, without new metastatic nodules, nodular interlobular septal thickening characteristic of lymphangitic carcinomatosis, or new pleural effusion, which excluded tumor progression and lymphangitic carcinomatosis as causes of respiratory deterioration. In summary, these findings excluded infectious, cardiogenic, thromboembolic, and neoplastic causes of respiratory deterioration. Bronchoscopy with BAL was not performed given the patient’s severe hypoxemia at presentation. After discussion by the lung cancer multidisciplinary team, the diagnosis was gefitinib-induced grade 3 ILD (CTCAE 5.0). At the time of ILD diagnosis, the patient’s ECOG performance status had deteriorated to 2.

Gefitinib was promptly discontinued, followed by the initiation of intravenous methylprednisolone (1.6 mg/kg/day), concomitant with supplemental oxygen therapy via nasal cannula. Seven days later, the patient experienced significantly improved cough and shortness of breath symptoms. The methylprednisolone dose was reduced to 0.8 mg/kg/day after week 4 and systematically tapered later to achieve complete discontinuation by week 8. On December 6, 2022, after approximately 8 weeks of methylprednisolone therapy, significant resolution of interstitial lung disease was observed on imaging (Fig. 2C), yet with a slightly increased size of the lung lesion (2.6 × 1.9 cm, Fig. 1C).

On December 6, 2022, due to adverse events associated with prior targeted therapy, the patient was transitioned to chemotherapy with pemetrexed (800 mg), carboplatin (AUC = 5), and bevacizumab (400 mg) to sustain antitumor treatment. The regimen was delivered over 6 cycles until April 25, 2023. During this period, the patient underwent a chest CT scan on February 28, 2023, which demonstrated a reduced pulmonary lesion measuring 1.9 × 1.5 cm (Fig. 1D), without radiological evidence of ILD (Fig. 2D). On April 25, 2023, the patient was given maintenance therapy with bevacizumab (400 mg) administered every 4 weeks, an interval selected based on the patient’s compliance and financial considerations during long-term maintenance treatment. After around 14 months, a follow-up CT scan on June 20, 2024 indicated significant tumor progression (3.3 × 2.8 cm, Fig. 1E), in the absence of ILD (Fig. 2E). The patient was initiated on antitumor therapy with docetaxel (100 mg) plus bevacizumab (400 mg). A total of 8 cycles of this combination regimen were administered throughout the treatment course: the first 6 cycles were delivered regularly at 4-week intervals, while the remaining 2 cycles were given intermittently with prolonged dosing intervals to further consolidate therapeutic efficacy, in consideration of docetaxel-related cumulative toxicity and the patient’s financial constraints. Bevacizumab was co-administered concurrently with docetaxel in all 8 treatment sessions, and no bevacizumab monotherapy maintenance was applied after the dosing adjustment. A follow-up chest CT scan on January 15, 2025 during treatment, demonstrated further tumor shrinkage (2.5 × 2.0 cm, Fig. 1F) and no signs of ILD (Fig. 2F). The overall combination treatment spanned approximately 10 months; repeat imaging on April 28, 2025 indicated disease progression (3.7 × 2.9 cm, Fig. 1G), again without evidence of ILD (Fig. 2G). At that time, the patient had an ECOG PS of 1, without obvious cough or dyspnea. Repeat molecular testing was performed using plasma ctDNA, which confirmed persistence of the EGFR exon 21 L858R sensitizing mutation and excluded newly-acquired resistance alterations. Repeat tissue biopsy was not performed because the patient declined further invasive procedures. Given the prior response to EGFR-TKI, the patient expressed a strong preference for rechallenge with EGFR-TKI therapy. After multidisciplinary team consultation, on April 28, 2025, the patient started rechallenge with oral almonertinib (110 mg daily), a third-generation EGFR-TKI, along with concomitant prophylactic methylprednisolone initiated at 0.4 mg/kg/day. The patient maintained stable respiratory status without cough, dyspnea, or other thoracic complaints, and peripheral oxygen saturation persistently exceeded 95% under ambient air (FiO_2_ = 21%). The tapering schedule of methylprednisolone was implemented stepwise as illustrated in Figure 3: the initial dose was maintained for 4 weeks, then reduced to 0.32 mg/kg daily. Thereafter, the dosage was decreased by 0.08 mg/kg every 2 weeks, reaching 0.24 mg/kg/day at week 6 and a low-dose maintenance dose of 0.16 mg/kg/day at week 8. At this most recent follow-up, this low-dose maintenance methylprednisolone (0.16 mg/kg/day) was still ongoing and had not been discontinued. No corticosteroid-related adverse events, including hyperglycemia, hypertension, gastrointestinal injury, or osteoporosis, were documented throughout the treatment course. Surveillance for recurrent ILD relied on daily home pulse oximetry and weekly telephone interviews focusing on respiratory symptoms including cough, dyspnea, and hypoxemia. The patient was instructed to discontinue almonertinib and seek immediate medical attention if fever, worsening cough, new-onset dyspnea, or oxygen desaturation occurred. Pulmonary function testing was not routinely performed in this patient. Subsequent follow-up confirmed the absence of cough, dyspnea, and other respiratory symptoms in the patient. At the most recent assessment on December 20, 2025, the patient received a chest CT, demonstrating tumor regression (2.5 × 2.2 cm, Fig. 1H), with no radiological recurrence of ILD (Fig. 2H). At follow-up, the patient reported no remarkable adverse events and voiced gratification over effective tumor suppression following TKI rechallenge without the recurrence of interstitial lung disease.

**Figure 3.**
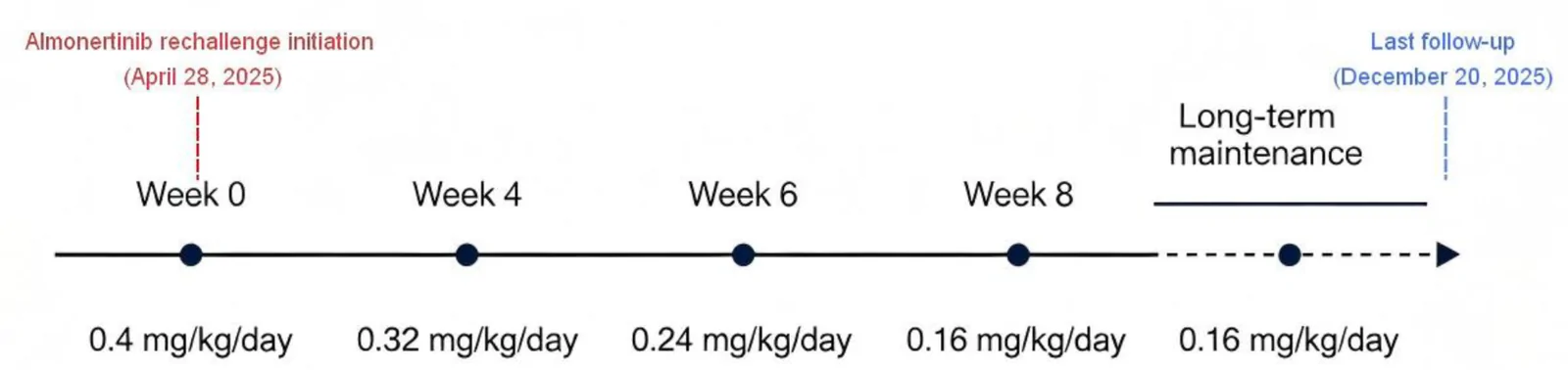
Stepwise tapering schedule of prophylactic methylprednisolone.

In our study, all procedures were performed according to the ethical principles outlined in the latest edition of the Declaration of Helsinki. Formal ethical approval was waived by our institutional ethics committee, as this retrospective single-case report only summarizes routine clinical care and does not qualify as human subject research. Written informed consent was obtained from the patient for participation and for the publication of the clinical details and radiographic images. All patient identifiers have been fully anonymized. Figure 4 illustrates the key diagnostic and therapeutic milestones of this case in a timeline format. The complete sequential systemic treatment regimen of the patient was systematically sorted and summarized for intuitive viewing (Table S1, Supplemental Digital Content 1).

**Figure 4.**
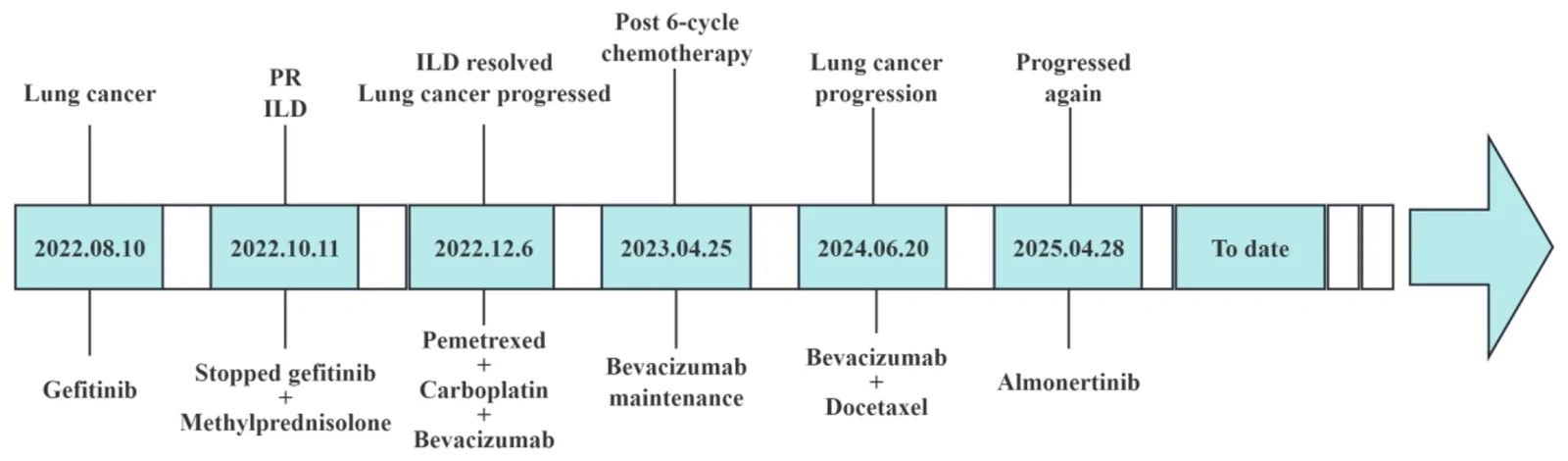
The timeline summarizing the key clinical events of this case.

## 3. Discussion

EGFR-TKIs have been documented to possess survival benefits and a potentially favorable tolerability profile, which are recommended as first-line therapy for patients harboring EGFR-sensitive mutations.^[4]^ However, EGFR-TKIs are associated with a distinct spectrum of adverse events, including rash, diarrhea, paronychia, oral mucositis, hepatotoxicity, especially ILD.^[9,10]^

ILD comprises a heterogeneous group of disorders characterized by inflammation and fibrosis of the lung parenchyma.^[11]^ EGFR-TKI–induced ILD, despite being clinically rare, is potentially fatal.^[12]^ Established risk factors for EGFR-TKI-associated ILD include male sex, recent radiotherapy or chemotherapy, smoking history, age ≥ 55 years, performance status score > 2, radiologically assessed normal lung parenchyma occupying <50%, preexisting ILD, coexisting emphysema or chronic obstructive pulmonary disease, concurrent pulmonary infection, short interval from cancer diagnosis (< 6 months), and comorbid cardiovascular disease.^[13]^ So far, there is a poor understanding of the mechanism underlying EGFR-TKI-induced ILD. Generally, members of the epidermal growth factor family have been proposed to contribute to pulmonary repair.^[14]^ Therefore, EGFR-TKI-mediated inhibition of EGFR signaling may impair bronchioloalveolar epithelial regeneration, thereby exacerbating lung injury. Given the essential role of EGFR in epithelial maintenance, EGFR-TKIs may disrupt epithelial proliferation and migration while dysregulating cytokine expression, consequently stimulating inflammatory cell infiltration and resultant parenchymal injury.^[6]^ Additionally, there may be a potential immune-mediated component in its pathogenesis given the clinical efficacy of corticosteroids in managing EGFR-TKI-related ILD.^[15]^ Building on this mechanistic framework, the divergent pulmonary toxicity profiles of first- and third-generation EGFR-TKIs may be further explained by differences in target selectivity. First-generation agents such as gefitinib reversibly inhibit both mutant and wild-type EGFR; since wild-type EGFR on alveolar type II epithelial cells mediates epithelial repair, its inhibition may compromise lung barrier integrity and contribute to ILD. In contrast, third-generation TKIs like almonertinib exhibit higher selectivity for mutant EGFR and weaker activity against wild-type EGFR, potentially reducing the risk of impaired epithelial repair.

EGFR-TKI-induced ILD shows typical manifestations of dry cough, progressive dyspnea, and fever. In patients receiving EGFR-TKIs with aggravated dyspnea, ILD should be suspected promptly, combined with urgent pulmonary imaging.^[16]^ In this context, EGFR-TKIs must be discontinued immediately upon suspicion or confirmation of ILD, with a timely initiation of appropriate therapeutic interventions required.

According to the Chinese Expert Consensus on the Management of Adverse Reactions of EGFR-TKIs, rechallenge with EGFR-TKIs should be avoided following the onset of EGFR-TKI-related ILD until the resolution of pulmonary injury. Patients with ≥ grade 3 ILD are recommended to permanently discontinue EGFR-TKIs. For those with grade 1–2 ILD, a cautious rechallenge may be considered only after complete resolution or marked improvement of interstitial lung abnormalities, provided that a favorable benefit-risk balance is identified through a comprehensive assessment and no alternative systemic therapies are available. Close clinical and radiological monitoring is mandatory during any subsequent treatment. Permanent discontinuation of EGFR-TKIs is required in the case of ILD recurrence upon re-exposure. Nevertheless, emerging evidence from limited studies suggests that rechallenge with EGFR-TKIs after recovery from ILD may still yield favorable clinical outcomes in selected cases.^[9]^ A broad overview of published experiences is provided in Table S2, Supplemental Digital Content 2, where the key baseline and clinical features of previously published EGFR‑TKI rechallenge cases after EGFR‑TKI‑related ILD are summarized. Notably, cases describing almonertinib rechallenge after grade 3 gefitinib‑induced ILD are very uncommon among these reports. Reported rechallenge strategies include^[17]^: continuation of the original EGFR-TKI at a reduced dose; rechallenge with an alternative EGFR-TKI in combination with corticosteroid administration; or rechallenge with another EGFR-TKI without corticosteroid administration. Longqiu Wu et al first reported a successful case of almonertinib rechallenge following osimertinib-induced ILD.^[18]^ Meanwhile, registrational phase III trials have demonstrated a low incidence of treatment-related interstitial lung disease (ILD) with almonertinib. In the phase III AENEAS trial enrolling only Chinese EGFR-mutant NSCLC patients, treatment-related ILD occurred in merely 0.9% of almonertinib recipients, all of which were grade 2 without grade ≥ 3 events.^[19]^ By contrast, the Asian subset of the FLAURA Phase III trial reported a 6% ILD incidence in patients treated with osimertinib.^[20]^ Indirect cross-trial comparisons imply that almonertinib may confer a more favorable pulmonary safety profile in East Asian populations, while prospective head-to-head randomized controlled trials are still lacking to confirm this distinction. In our case of grade 3 gefitinib-induced ILD, the patient was successfully transitioned to almonertinib treatment after full recovery of interstitial lung injury and an interim period of chemotherapy and anti-angiogenic therapy.

Almonertinib is a novel third-generation EGFR-TKI that has been approved for treating NSCLC patients harboring EGFR-sensitizing mutations. In this case, the patient developed severe ILD during gefitinib treatment and received 6 cycles of platinum-based chemotherapy after discontinuation of this drug, followed by maintenance therapy with an anti-angiogenic agent. Upon disease progression, the patient was provided with combination therapy using an anti-angiogenic agent and an alternative chemotherapeutic regimen. Nevertheless, the patient experienced further tumor progression, without other viable systemic treatment options. Immunotherapy was discarded given its limited efficacy in patients with isolated EGFR-mutant NSCLC, the potential risk of exacerbating prior grade 3 ILD, and lack of local medical insurance reimbursement. As a prefecture-level medical institution, our hospital had no ongoing clinical trials recruiting patients with advanced EGFR-mutant NSCLC. In addition, nationwide relevant trial protocols uniformly excluded individuals with a history of grade ≥ 3 TKI-associated ILD, leaving no eligible trial options for this patient. Extensive pleural and mediastinal metastases rendered curative ablation and SBRT unfeasible, whereas palliative thoracic radiotherapy was ruled out owing to the high risk of superimposed radiation pneumonitis. Taken together, no alternative antitumor regimens were available.

A dedicated lung cancer MDT meeting was convened to comprehensively evaluate the risks and benefits of EGFR-TKI rechallenge. On the risk side, authoritative consensus guidelines advise permanent TKI discontinuation following grade ≥ 3 ILD, since rechallenge carries a theoretical risk of recurrent severe interstitial lung injury. On the benefit side, the tumor exhibited a marked response to initial gefitinib therapy; real-world data from Chinese cohorts demonstrate a lower incidence of ILD with almonertinib, and published case reports validate the prophylactic anti-inflammatory effect of low-dose corticosteroids.

Multiple objective indicators confirmed substantial recovery of pulmonary lesions and satisfactory physical tolerance prior to rechallenge: The patient had an ECOG performance status of 1 and could complete all daily activities independently without resting dyspnea; Serial chest CT scans acquired by April 28, 2025 showed prominent resolution of bilateral interstitial infiltrates, with no evident residual fibrosis or honeycomb changes.

After thorough counseling covering all potential pulmonary adverse events, she repeatedly requested EGFR-TKI rechallenge, and written informed consent for this off-label rechallenge was obtained. The MDT group reached a unanimous consensus that the antitumor benefits outweighed pulmonary toxic risks under stringent clinical and radiological surveillance, and the combination regimen of almonertinib plus methylprednisolone was approved. Almonertinib was selected owing to its high selectivity for mutant-type EGFR, with indirect cross-trial observations suggesting a comparatively low rate of TKI-related ILD in East-Asian cohorts, and proven antitumor activity against the patient’s EGFR-sensitizing alteration.

At the last follow-up, the patient had been on almonertinib for approximately 8 months, with sustained tumor shrinkage on radiological assessment, with no presence of ILD recurrence or other significant adverse events. It should be noted that the absence of recurrent ILD in this case cannot be solely attributed to almonertinib, since prophylactic systemic corticosteroids were administered concomitantly throughout the rechallenge period.

To the best of our knowledge, published cases of almonertinib rechallenge after EGFR-TKI-induced ILD remain scarce, mostly reported in the context of osimertinib-related ILD. Cases of almonertinib rechallenge following grade 3 gefitinib-induced ILD are particularly rare, and the present case enriches this limited but clinically relevant evidence base. Subgroup data from the Reiwa study further support the rationale for this strategy: among patients rechallenged with EGFR-TKI after drug-related ILD, the overall recurrence rate was 16% (5/31), with a 28% recurrence rate (5/18) in the osimertinib same-drug rechallenge group and zero recurrence (0/13) in patients rechallenged with an alternative EGFR-TKI.^[21]^

However, this study has several limitations. First, as a single-case report, the findings have inherently limited generalizability. The favorable outcome in this patient does not confirm the safety of almonertinib rechallenge for all patients with prior EGFR-TKI-induced ILD, and individual recurrence risk remains unpredictable. Second, prophylactic corticosteroids were co-administered with almonertinib throughout the rechallenge period; therefore, the contribution of corticosteroid prophylaxis to the absence of ILD recurrence cannot be separated from the effect of almonertinib rechallenge. The favorable outcome may reflect the combined effect of corticosteroid coverage and the pulmonary safety profile of almonertinib, rather than the effect of almonertinib alone. Third, the absence of bronchoscopy and bronchoalveolar lavage (BAL) represents a limitation of the diagnostic workup for drug-induced ILD, as invasive pulmonary sampling was not performed to further exclude atypical infectious or inflammatory etiologies. Fourth, the mechanisms underlying the differential pulmonary toxicity between gefitinib and almonertinib remain incompletely understood. Larger prospective studies are needed to further validate the safety and optimal patient selection for third-generation EGFR-TKI rechallenge after drug-induced ILD.

## 4. Conclusion

For patients with ≥ grade 3 EGFR‑TKI‑related ILD, EGFR‑TKI re‑challenge is typically not advised owing to the high risk of recurrent ILD. Data from this single case represent a hypothesis‑generating observation rather than a general therapeutic recommendation. For highly‑selected patients with progressive disease and no available antitumor alternatives, carefully‑monitored almonertinib rechallenge combined with corticosteroids may be considered as a tentative option. Multiple factors may have contributed to this favorable outcome, and validation in larger cohorts is still required.

## Author contributions

**Visualization:** Jie Liu.

**Writing – original draft:** Jie Liu.

**Writing – review & editing:** Jie Liu, Yunhu Pan.

**Investigation:** Tengchen Wei, Zhenming Bao, Peng Lai.

**Supervision:** Tengchen Wei, Zhenming Bao, Fangfang Huang, Yunhu Pan.

**Validation:** Tengchen Wei.




