# Supplementary material for: Successful rechallenge with almonertinib and corticosteroids after gefitinib-induced interstitial lung disease: A case report and literature review
Source: Medicine (Baltimore). 2026 Nov 6;105(45):e51470. doi: 10.1097/MD.0000000000051470 (PMC13747485; doi:10.1097/MD.0000000000051470)
Supplement: Supplementary file 1 [file medi-105-e51470-s001.docx]

| **Treatment phase** | **Date of initiation** | **Date of discontinuation** | **Duration of treatment** | **Regimen details** |
| --- | --- | --- | --- | --- |
| 1st-line EGFR-TKI (Gefitinib) | Aug 10, 2022 | Oct 11, 2022 | ~2 months | Gefitinib 250 mg qd |
| ILD rescue steroid therapy | Oct 11, 2022 | Dec 06, 2022 | ~8 weeks | Methylprednisolone, gradual tapering |
| Platinum-based induction chemotherapy + anti-angiogenesis | Dec 06, 2022 | Apr 25, 2023 | 6 cycles | Pemetrexed + carboplatin (AUC=5) + bevacizumab 400 mg q4w |
| Bevacizumab maintenance monotherapy | Apr 25, 2023 | Jun 20, 2024 | ~14 months | Bevacizumab 400 mg q4w |
| Second-line chemotherapy + anti-angiogenesis | Jun 20, 2024 | Apr 28, 2025 | ~10 months | Docetaxel + bevacizumab |
| 3rd-generation EGFR-TKI rechallenge + prophylactic steroid | Apr 28, 2025 | Dec 20, 2025 (last follow-up) | ~8 months | Almonertinib 110 mg qd + low-dose methylprednisolone (stepwise tapering) |
