# Supplementary material for: Successful rechallenge with almonertinib and corticosteroids after gefitinib-induced interstitial lung disease: A case report and literature review
Source: Medicine (Baltimore). 2026 Nov 6;105(45):e51470. doi: 10.1097/MD.0000000000051470 (PMC13747485; doi:10.1097/MD.0000000000051470)
Supplement: Supplementary file 2 [file medi-105-e51470-s002.docx]

| **Case** | **Age/sex** | **EGFR mutation** | **Drug dose (mg)** | **Time to onset, days** | **ILD grade** | **Rechallenge drug (mg)** | **CS therapy** | **Recurrence of ILD** | **Reference** |
| --- | --- | --- | --- | --- | --- | --- | --- | --- | --- |
| 1 | 56/M | L858R | GEF 250 | 45 | 3 | GEF 250 | NO | NO | 1 |
| 2 | 59/M | NA | GEF 250 | 23 | 3 | GEF 250 | NO | YES | 2 |
| 3 | 28/F | Exon 19 deletion | GEF 250 | 28 | 3 | ERL 50-75 | PSL 30 | NO | 3 |
| 4 | 77/F | NA | GEF 250 | 49 | 2 | ERL 100 | PSL 10 | NO | 4 |
| 5 | 62/M | Exon 19 deletion | GEF 250 | 24 | 3 | ERL 150 | NO | NO | 5 |
| 6 | 41/F | NA | GEF 250 | 20 | 3 | ERL 75 | PSL 20 | NO | 6 |
| 7 | 56/F | Exon 19 deletion | ERL 150 | 35 | 2 | ERL 50-75 | PSL 40 | NO | 7 |
| 8 | 68/F | L858R | ERL 150 | 56 | 2 | GEF 250-ERL 150 | BMT 4 | NO | 8 |
| 9 | 62/F | Exon 19 deletion | GEF 250 | 29 | 2 | ERL 25-50 | NO | YES | 9 |
| 10 | 49/F | Exon 19 deletion | GEF 250 | 2 | 2 | ERL 150 | NO | NO | 10 |
| 11 | 38/F | L858R | OSI 80 | 31 | NA | OSI 80 | NO | NO | 11 |
| 12 | 75/F | Exon 19 deletion | OSI 80 | 64 | 2 | OSI 40 | PSL 0.5 mg/kg | NO | 12 |
| 13 | 62/M | Exon 19 deletion | OSI 80 | 82 | 2 | OSI 40 | PSL 25 | NO | 13 |
| 14 | 69/F | L858R | OSI 80 | 55 | 1 | OSI 40 | PSL 10 | NO | 14 |
| 15 | 57/F | Exon 19 deletion | OSI 80 | 21 | 3 | OSI 80 | PSL 0.5 mg/kg | NO | 15 |
| 16 | 75/F | Exon 19 deletion | OSI 80 | 180 | 2 | OSI 80 | PSL 5 | NO | 16 |
| 17 | 71/F | Exon 19 deletion | OSI 80 | 7 | 3 | OSI 40 | NO | NO | 17 |
| 18 | 78/F | L858R | OSI 80 | 135 | 2 | AFA 30 | NO | NO | 18 |
| 19 | 84/F | Exon 19 deletion | OSI 80 | 46 | 3 | AFA 20 | PSL 5 | NO | 19 |
| 20 | 81/F | L858R | OSI 80 | 44 | 3 | ERL 100 | PSL 5 | NO | 20 |
| 21 | 62/F | Exon 19 deletion | OSI 80 | 69 | 3 | GEF 250 | PSL 5 | NO | 21 |
| 22 | 82/F | Exon 19 deletion | OSI 80 | 49 | 3 | GEF 250 | PSL 15 | NO | 21 |
| 23 | 77/M | L858R | OSI 40 | 7 | 2 | GEF 250 | NO | YES | 21 |
| 24 | 72/F | L858R | OSI 80 | 62 | 3 | GEF 250 | NO | NO | 21 |
| 25 | 57/F | L858R | OSI 80 | 15 | 2 | GEF 250 | PSL 30 | NO | 21 |
| 26 | 78/F | L858R | OSI 80 | 51 | 3 | GEF 250 | NO | NO | 21 |
| 27 | 67/F | L858R | OSI 80 | 42 | 2 | AFA 20 | PSL 10 | NO | 22 |
| 28 | 67/F | L858R | OSI 80 | 42 | 2 | OSI 40 | PSL 20 | NO | 22 |
| 29 | 56/F | Exon 19 deletion | OSI 80 | 96 | 2 | FUR 80 | PSL 20 | NO | 23 |
| 30 | 73/M | Exon 19 deletion | OSI 80 | 93 | 4 | ALM 55-110 | NO | NO | 24 |
| 31 | 62/F | L858R | OSI 80 | 90 | 2 | ALM 110 | NO | NO | 25 |
| 32 | 64/F | L858R | OSI 80 | 90 | 2 | ALM 55 | PSL 10 | NO | 26 |
| 33 | 76/F | Exon 19 deletion | OSI 80 | 60 | 3 | ALM 110 | NA | NO | 27 |

**REFERENCES**

**1.**	Takamochi K, Suzuki K, Bashar AH, et al. Readministration of gefitinib in a responder after treatment discontinuation due to gefinitib-related interstitial lung disease: a case report. *J Med Case Rep*. 2007;1:138.

**2.**	Suzuki M, Asahina H, Konishi J, Yamazaki K, Nishimura M. Recurrent gefitinib-induced interstitial lung disease. *Intern Med*. 2008;47(6):533-536.

**3.**	Fukui T, Otani S, Hataishi R, et al. Successful rechallenge with erlotinib in a patient with EGFR-mutant lung adenocarcinoma who developed gefitinib-related interstitial lung disease. *Cancer Chemother Pharmacol*. 2010;65(4):803-806.

**4.**	Chang SC, Chang CY, Chen CY, Yu CJ. Successful erlotinib rechallenge after gefitinib-induced acute interstitial pneumonia. *J Thorac Oncol*. 2010;5(7):1105-1106.

**5.**	Takeda M, Okamoto I, Makimura C, Fukuoka M, Nakagawa K. Successful treatment with erlotinib after gefitinib-induced severe interstitial lung disease. *J Thorac Oncol*. 2010;5(7):1103-1104.

**6.**	Tian Q, Chen LA. Erlotinib achieved partial response in a non-small cell lung cancer patient with gefitinib-induced interstitial lung disease. *Case Rep Oncol*. 2011;4(3):464-466.

**7.**	Dallas JL, Jantz MA, Lightsey JL, Sonntag C, Kaye FJ. Successful erlotinib rechallenge after erlotinib-induced interstitial lung disease. *J Thorac Oncol*. 2011;6(6):1142-1143.

**8.**	Togashi Y, Masago K, Hamatani Y, et al. Successful erlotinib rechallenge for leptomeningeal metastases of lung adenocarcinoma after erlotinib-induced interstitial lung disease: a case report and review of the literature. *Lung Cancer*. 2012;77(2):464-468.

**9.**	Arakawa N, Tsujita A, Saito N, Ishikawa S, Ohno S. Successful erlotinib rechallenge after both gefitinib- and erlotinib-induced interstitial lung diseases. *Respirol Case Rep*. 2013;1(1):17-19.

**10.**	Nakamichi S, Kubota K, Horinouchi H, et al. Successful EGFR-TKI rechallenge of leptomeningeal carcinomatosis after gefitinib-induced interstitial lung disease. *Jpn J Clin Oncol*. 2013;43(4):422-425.

**11.**	Mamesaya N, Kenmotsu H, Takahashi T. Successful osimertinib rechallenge in a patient with advanced non-small cell lung cancer following osimertinib-induced interstitial lung disease after treatment with nivolumab. *Invest New Drugs*. 2017;35(6):839-841.

**12.**	Miyauchi E, Ichinose M, Inoue A. Successful Osimertinib Rechallenge in a Patient with T790M-Mutant Non-Small Cell Lung Cancer after Osimertinib-Induced Interstitial Lung Disease. *J Thorac Oncol*. 2017;12(5):e59-e61.

**13.**	Kiriu T, Tamura D, Tachihara M, et al. Successful Osimertinib Rechallenge with Steroid Therapy after Osimertinib-induced Interstitial Lung Disease. *Intern Med*. 2018;57(1):91-95.

**14.**	Satoh S, Shiroyama T, Tamiya M, et al. Successful osimertinib rechallenge after osimertinib-induced pneumonitis in a patient with lung adenocarcinoma. *Respir Med Case Rep*. 2018;23:68-70.

**15.**	Lu H, Dowell J. Osimertinib in Pulmonary Manifestations: Two Case Reports and Review of the Literature. *In Vivo*. 2020;34(1):315-319.

**16.**	Itano J, Higo H, Ohashi K, et al. Successful Re-administration of Osimertinib in Osimertinib-induced Interstitial Lung Disease with an Organizing Pneumonia Pattern: A Case Report and Literature Review. *Intern Med*. 2020;59(6):823-828.

**17.**	Mohammed T, Mangeshkar S, Rathmann J. Successful Rechallenge with Osimertinib after Very Acute Onset of Drug-Induced Pneumonitis. *Case Rep Oncol*. 2021;14(2):733-738.

**18.**	Nishima S, Miyanaga A, Saito S, et al. Successful Treatment with Afatinib after Osimertinib-induced Interstitial Lung Disease in a Patient with EGFR-mutant Non-small-cell Lung Cancer. *Intern Med*. 2021;60(4):591-594.

**19.**	Sato Y, Sekine A, Hagiwara E, et al. Successful treatment with afatinib following the failure of osimertinib rechallenge with osimertinib-induced interstitial lung disease: A case report. *Respir Med Case Rep*. 2021;33:101450.

**20.**	Fujimoto S, Katsurada N, Hazama D, Yamamoto M, Nagano T, Tachihara M. Successful Treatment with Ramucirumab Plus Erlotinib following Osimertinib-induced Interstitial Lung Disease. *Intern Med*. 2025;64(5):749-751.

**21.**	Shimbu K, Hisakane K, Kadoma N, et al. Safety and Efficacy of Gefitinib Administration After Osimertinib-Induced Interstitial Lung Disease: A Six-Case Series. *Onco Targets Ther*. 2024;17:717-726.

**22.**	Yanai M, Sakamoto T, Uetani N, et al. Successful Switch to Afatinib and Osimertinib Rechallenge with Corticosteroids after Osimertinib-induced Interstitial Lung Disease: A Case Report and Literature Review. *Intern Med*. 2025;64(16):2478-2483.

**23.**	Wei FF, Zhang J, Jia Z, Yao ZC, Chen CQ. Furmonertinib re-challenge for epidermal growth factor receptor-mutant lung adenocarcinoma after osimertinib-induced interstitial lung disease: A case report. *World J Clin Oncol*. 2025;16(3):101766.

**24.**	Bao H, Deng C, Luo X, et al. Case Report: A successful rechallenge with aumolertinib after osimertinib-induced severe interstitial lung disease. *Front Pharmacol*. 2026;17:1785616.

**25.**	Zhang J, Huang S, Shen H. Sequential almonertinib therapy following osimertinib-induced interstitial lung disease in early-stage EGFR-mutant lung adenocarcinoma: a case report. *Front Oncol*. 2026;16:1699804.

**26.**	Chen W, Zhang L, Shen H, Wang B, Luo J, Cui E. Successful administration of low-dose almonertinib in a patient with lung adenocarcinoma after osimertinib-induced interstitial lung disease: a case report and literature review. *Anticancer Drugs*. 2023;34(3):460-466.

**27.**	Wu L, Zhong W, Li A, et al. Successful treatment of EGFR T790M-mutant non-small cell lung cancer with almonertinib after osimertinib-induced interstitial lung disease: a case report and literature review. *Ann Transl Med*. 2021;9(11):950.
